# AI Personalization and Its Influence on Online Gamblers’ Behavior

**DOI:** 10.3390/bs15060779

**Published:** 2025-06-04

**Authors:** Florin Mihai, Ofelia Ema Aleca, Daniel-Marius Iordache

**Affiliations:** Management Information Systems Department, Faculty of Accounting and Management Information Systems, Bucharest University of Economic Studies, 010552 Bucharest, Romania; florin.mihai@cig.ase.ro (F.M.); ofelia.aleca@cig.ase.ro (O.E.A.)

**Keywords:** artificial intelligence, online gambling, user behavior, algorithmic personalization, gambling addiction, behavioral targeting, responsible gambling, predictive analytics

## Abstract

Technological advancements in algorithmic personalization are widely believed to influence user behavior on online gambling platforms. This study explores how such developments, potentially including AI-driven mechanisms, may affect cognitive and motivational processes, especially in relation to risk perception, decision-making, and betting persistence. Using ordinary least squares (OLS) and panel regression models applied to behavioral data from a gambling platform, we examine patterns that are consistent with increased personalization between two distinct time periods, 2016 and 2021. The datasets do not contain any direct metadata regarding AI interventions. However, we interpret changes in user behavior over time as indicative of evolving personalization dynamics within a broader technological and contextual landscape. Accordingly, our conclusions about algorithmic personalization are inferential and exploratory, drawn from temporal comparisons between 2016 and 2021. Our findings show that users receiving personalized bonuses or making early cash-out decisions tend to adjust their stake sizes and betting frequency in systematic ways, which may reflect indirect effects of technological reinforcement strategies. These behavioral patterns raise important ethical and regulatory questions, particularly regarding user autonomy, algorithmic transparency, and the protection of at-risk users. This research contributes to the literature on digital behavior influencing gambling by framing the analysis as observational and quasi-experimental and suggests that further studies use experimental and log-level data to more specifically analyze the algorithmic effects. However, no causal claims can be made about AI influence as the temporal contradictions are interpreted as broad phenomena of technological developments, since they are not measured as algorithmic interventions. Further studies should also investigate the development of predictive models aimed at countering gambling addiction; evaluate the long-term ethical implications of algorithmic personalization; and discuss potential solutions codeveloped to foster a responsible gambling climate.

## 1. Introduction

The online gambling sector has seen considerable growth and development over the past ten years due to technological advancements and greater internet access. Technology has become a critical driver of this expansion, enabling personalized incentives, adaptive interfaces, and behavioral tracking. Technology improves the player experience by providing tailored promotions, personalized retention strategies, and tracking player betting behavior. Despite technology’s increasing role in gambling platforms, its impact on user cognition and decision-making remains insufficiently understood. 

Although technologies and practices related to personalization (potentially including AI) are becoming increasingly commonplace in gambling, this research does not describe or isolate algorithmic interventions as part of its observations. Instead, temporal changes in behaviors of users are examined as patterns that we might expect to observe in relation to a broader development of technology and contexts as they changed qualitatively between 2016 and 2021. As such, this study is methodologically framed as observational and quasi-experimental, relying on temporal comparisons (i.e., between 2016 and 2021) as an indirect proxy for the evolution of personalization technologies. Any inference regarding AI influence is therefore conceptual and not based on intervention-specific data.

This observational design does not allow us to isolate specific algorithmic interventions. Therefore, all references to AI-related mechanisms should be understood as conceptual and based on temporal inference, rather than on direct system logs or platform-side documentation. This is especially the case for how algorithms influence users’ risk perceptions, the user’s sense of control, and betting strategies.

Recent research shows that technology can leverage certain cognitive mechanisms to influence decisions. For instance, personalization algorithms may reinforce the illusion of control—the belief that one’s actions can change the outcome—and strengthen loss aversion, prompting users to continue betting to recoup losses (M. M. Auer & Griffiths, 2015; van Holst et al., 2014). Moreover, by analyzing each player’s behavior, AI can anticipate moments of emotional vulnerability and provide personalized stimuli just when users are most likely to keep betting (Gainsbury et al., 2017). From a behavioral-science standpoint, these personalized reinforcements reproduce a classic operant-conditioning schedule, in which variable-ratio rewards strengthen the persistence of the target behavior (Skinner, 1938). This dynamic adaptation of interactions can encourage compulsive behavior and extend the time spent on the platform (Poudel et al., 2024; Wong et al., 2023).

In practice, AI-enabled personalization on gaming sites manifests in several observable actions that can affect how users experience content. For example, once a specific algorithm sees that a player is about to log off or that the player has just experienced a losing streak, the platform may present a bonus offer. The express delivery of the offer will often come in the form of personalized messages, free credits, or limited-time promotions, which provide a monetary incentive for users to return to the platform. In non-AI systems, these bonuses are generic and consistent. However, AI systems allow for bonus offers to evolve from the specific combination of timing, value, and content to the unique profile of behavior associated with each user. Moreover, users can also receive betting options and payout suggestions in real time, as the AI adapts to each user’s previous bets in an attempt to incite risk-taking through framing effects, even when such reframing is imperceptible. These interactions cannot help but be invisible to the user; however, the interaction does change the psychology of decision-making on a different level than in non-AI systems which only involve predetermined outcomes and static offer propositions.

Another important area is personalized financial incentives and intermittent reward systems. Research suggests gambling sites use sophisticated algorithms to deliver personalized bonuses and promotions with the intention of enhancing player engagement. This is similar to social media and video gaming, where artificial intelligence creates a repetitive cycle of anticipation and reward that can lead to conditioned psychological dependence (Clark & Zack, 2023). However, the extent to which these mechanisms affect players’ risk perception and perceived odds of winning remains unclear, as does the influence AI’s evolution has had on these processes over time.

In addition to gain-related motives, there is substantial literature that highlights gambling as a means of emotional escape when experiencing stress, anxiety, or negative affect. Avoidance gambling is a much stronger predictor of gambling disorder compared to simply experiencing the motivation of wanting to win money (Alaba-Ekpo et al., 2024; Flack & Morris, 2015; Marchica et al., 2020). Participants who gamble to dissociate or manage their mood may be especially prompted by AI interventions, as algorithmic feedback loops can evidence further compulsive behavior amid emotional turmoil. While our dataset cannot enable us to assess user motivations directly, we recognize it as an important distinction and incorporate this interpretation into our review of behavioral outcomes. In the future, research should consider identifying psychological profiles to differentiate gain-seeking from escape-oriented gambling paths. 

Online gambling has grown to be an increasingly important part of the overall gambling sector. By 2021, the online segment had matured into a major share of global gambling, generating an estimated USD 102 billion in revenue. The United Kingdom was the single largest market, accounting for about USD 12.48 billion, followed by the United States and Australia (Junaedi, 2024). Europe as a whole has long been the epicenter of digital betting: it had already captured roughly 42 percent of the worldwide online-gambling revenue in 2018 (Polyzoidis, 2019) and, within the European market itself, online stakes grew from EUR 24.5 billion in 2019 to a projected one-third of total gambling turnover by 2025 (Ivanov, 2021). The COVID-19 pandemic created further momentum in this regard, temporarily shifting consumer behavior toward digital formats. Additionally, the infusion of new technologies, particularly the use of AI-driven features such as real-time personalization and adaptive reward structures, has changed online gambling to be more interactive and accessible than at any time in the past. In light of this, understanding behavioral patterns related to gambling in this space is increasingly imperative—particularly as projections indicate that the expansion of online and hybrid gambling models has the potential to impact the overall general gambling ecosystem.

This research examines the degree to which algorithmic-driven personalization affects gambling behaviors on digital gambling sites, through five identifiable and inter-related objectives. First, we will evaluate the degree to which algorithmic tailoring alters perceptions of risk and investment behavior in relation to betting behavior, which may be demonstrated through changes to stake amounts, every betting frequency, and moment-to-moment decision-making. Second, we will assess gambling behavior from two distinct moments in time—late 2016 and spring 2021—to better understand how cumulative generations of engagement algorithms have altered gambling behavior and user retention, over time. Third, we will assess the mechanics of changing behaviors, asking how targeted bonuses and promotional content influence attentiveness during the session, and the subjective calculation of likelihood to keep playing. Fourth, we will evaluate the extent to which algorithms may have changed cash-out decisions, or at least encouraged a player to keep their funds on-site and maintain an attentional feedback loop that prolongs gambling behavior. Lastly, we will consider the ethical and regulatory implications, identifying the behavioral risks co-created through large-scale personalization, while advocating for protection for vulnerable users.

Despite previous research focusing on fraud detection and user retention, the more general behavioral implications of AI-driven personalization in online gambling contexts are relatively unknown. This is relevant as we are beginning to assess how algorithmic systems impact users’ understanding of risk, control, and decision-making over time. There are few empirical studies measuring changes in gambling behavior corresponding to changing AI mechanisms—especially those using real world data tracking behavior as technology evolves.

We apply statistical procedures to illustrate how AI alters the cognitive processes guiding players’ strategies and decision-making, showing that AI not only engages and retains players but also influences their perceptions and choices. We present data suggesting that AI has become more effective at conditioning users’ perceived odds of winning and control over the game—a development that is very concerning from an ethical perspective.

Consequently, the study expands prior research by presenting a different insight into the impact of AI in gambling, exemplifying the effects of intermittent reward schedules on user behavior. It also provides an empirical basis for policymaking aimed at protecting individuals vulnerable to gambling addiction.

The methodology includes descriptive statistical analyses and OLS regression models to observe behavioral patterns and to determine whether and how algorithms influence betting behavior. 

This approach provides perspective in three critical ways:

- Behavioral psychology, through its analysis of the influence of artificial intelligence on users’ risk profile and anticipated rewards.
- Human–computer interaction, through its analysis of the impact of algorithmic personalization on decision-making.
- AI ethics and regulation will be examined by exposing the behavioral risks associated with AI-enabled gambling models and suggest regulatory directions.

Accordingly, we advance the following working hypotheses:

**H1.**  *Players who cash out early place larger stakes per session.*

**H2.**  *Players who receive a bonus place smaller stakes per session.*

**H3.**  *Higher profit is associated with larger stakes per session.*

**H4.**  *Receiving a bonus reduces the number of consecutive bets.*

**H5.**  *Higher profit is associated with fewer consecutive bets.*

**H6.**  *Early cash-out behavior is linked to fewer consecutive bets.*

**H7.**  *The strength and/or direction of the above associations changes between 2016 and 2021.*

The concept of bounded rationality (Simon, 1955) theoretically underpins these hypotheses. It follows that after achieving an adequate outcome, for example, making a profit or cashing out early, players intentionally choose to reduce their risk exposure. This is a satisficing strategy and is consistent with recent research in gambling behavior pertaining to goal completion, impulse control, and emotion regulation (Marchica et al., 2020; M. Auer & Griffiths, 2022). Users may self-regulate by betting less frequently in light of their perceived success or emotional state, thereby reducing the chances of an escalation of compulsive behavior.

Likewise, previously published empirical work suggests that framing effects and the anticipation or expectation of a reward can affect user responses to personalized bonuses, perceived profitability, and real-time incentives in betting that determine both wager size and frequency. Temporal or temporal comparisons may also expose changes in such behaviors, not based on an isolated causal effect of the user functioning in the technology environment but based on an anticipation of a change or improvement in technology features over time or history. 

Instead of providing a singular causal explanation, this study contributes to theoretical discussions by illustrating how personalization technologies (which likely include artificial intelligence) can amplify familiar cognitive and emotional biases in gambling (e.g., loss aversion, the illusion of control, and satisfaction). Our focus is on how these mechanisms potentially behave organically after spending time with the personalization technology, with the ongoing refinements potentially leading to more sophisticated personalization designs.

## 2. Theoretical Framework

### 2.1. AI Algorithms in Gambling: Shaping and Influencing User Decisions

Recent research stresses that AI has a vital role in shaping user behavior on online gambling platforms, directly via interfaces and algorithms, along with indirect social and financial mechanisms. For example, Wong et al. (2023) show that transparent and visible usage of AI on the interface, combined with social influences and financial rewards, greatly boosts users’ intention to place bets. In a bibliometric work by Iordache et al. (2024) data confirms a significant rise in research focused on gambling risks and digital technologies. Analyzing nearly a thousand publications over two decades, they show that algorithmic personalization and user behavior prediction are now core themes in gambling studies, reinforcing the relevance of AI-focused behavioral research.

In parallel, Sirola et al. (2021) demonstrate how AI algorithms on social media are salient in promoting gambling content, arguing that AI-driven recommendations and social norms significantly influence user behavior in these environments, especially through younger audiences. M. Auer and Griffiths (2022) further explore AI’s potency in predicting user behavior concerning financial limit-setting in betting. They accurately anticipated how users manage their gambling budgets using machine-learning algorithms, a key insight that can help identify problematic behaviors in an early phase.

Studies on online gambling behavior, such as (Sirola et al., 2021), also underscore prior outcomes and accumulated experience as influencing user decisions. Their findings showcase a direct and profound influence of previous results on future strategies and choices. Whiteford et al. (2022) also affirm that predictability in algorithmic interfaces can affect users’ sense of control, in turn affecting their betting behavior. They found that users adjust their betting strategies when employing interfaces that enhance their perceived control, impacting both the amount and frequency of placed bets.

In this regard, AI affects user behavior in the gambling domain by shaping user perceptions and influencing emotional responses, assisting with social dynamics.

Given AI’s significant role in shaping user perceptions, it is also important to examine the underlying cognitive and emotional mechanisms that drive gambling behavior. Yet, we emphasize that we do not directly observe or isolate algorithmic interventions. The presumed role of personalization technologies is inferred from contextual and temporal behavioral patterns, not from platform-side metadata. Accordingly, references to algorithmic personalization in this study are conceptual and grounded in the existing literature, serving as a secondary lens for interpreting observed user behaviors.

Also, we do not know the exact ways the platform used personalized strategies. Our analysis is based on publicly available databases that do not include internal system logs or algorithmic delivery dumb traces. Therefore, any reference to AI-related mechanisms is theoretical interpretation and not actual measurement.

Throughout this paper, we use the term “AI personalization” to refer to general platform-level engagement strategies that may involve algorithmic components. These references are interpretative in nature and based on literature-informed assumptions and behavioral comparisons between 2016 and 2021.

### 2.2. Cognitive and Emotional Mechanisms in Gambling: From Impulsivity to Digital Dependency

Psychological factors are crucial for understanding gambling behavior. The literature indicates that neurobiological and psychological mechanisms—such as processing rewards, near-miss effects, and the cognitive mechanism of decision-making—are all connected with addictions (Mattson et al., 2008; Mestre-Bach et al., 2018). Impulsivity often goes hand in hand with problematic or even criminal behavior in regard to pathological gamblers, thus suggesting the greater vulnerability of that group (Mestre-Bach et al., 2018).

Beyond individual traits like impulsivity, gambling behavior is driven by a complex motivational structure of gambling, which is multifaceted and can be conceptualized as comprising five dimensions: socialization, pursuit of entertainment, avoidant behavior, emotional arousal, and financial gain. Of these, profit-seeking often accompanies incipient gambling problems (Lee et al., 2007). In online gambling, financial motivations prevail, and perceiving gambling as a potential income stream can put individuals at a much higher risk of pathological behavior (Dennis et al., 2017; Lee et al., 2007).

Furthermore, these motivational factors are closely intertwined with psychological conditions—for example, anxiety and depression can directly influence why individuals gamble—suggesting that responsible gambling interventions may be designed accordingly to suit these individual motivations and symptoms (Dennis et al., 2017). Poor self-efficacy in gambling behavior management and distorted views of winnings in gambling can further raise the risk of pathological dependence, especially vulnerable populations such as older adults (M. Auer & Griffiths, 2022; Dennis et al., 2017).

An analysis of these psychological factors underscores the importance of understanding the cognitive and emotional mechanisms underlying gambling, which ultimately guide the development of effective prevention and treatment strategies (Murch et al., 2023).

One direct outgrowth of these cognitive and emotional mechanisms is the concept of digital addiction in gambling coined by (Parveen et al., 2024). Digital addiction is a term used for activities like online gambling and overuse of smartphones and is largely explained by psychological mechanisms involving reward and reinforcement (Parveen et al., 2024). Redish (2004) argues that addictions arise from a dysfunction within a reward and learning system where reinforcement processes-normally intended to help individuals adapt their behavior-become impaired and fortify addictive patterns. This proposition has also been supported in the case of smartphone use; two major mechanisms have been identified through which digital addiction originates: immediate rewards and the setting up of automatic habits (Chen et al., 2019). These two are interrelated, such that they together act to augment compulsive behaviors by continually reinforcing user engagement on the digital platforms. 

In addition to reinforcement-driven habits associated with digital addiction, cognitive biases in how users perceive risk and reward also play a crucial role. One study on individuals’ dispositions toward losses and gains found that losses provoke a much stronger reaction than gains (Ruggeri et al., 2020). This propensity is better explained with the aid of Cumulative Prospect Theory; individuals are somewhat inclined to take chances when they stand chances of making up for losses (Ruggeri et al., 2020). AI-balanced algorithms exploit the cognitive and behavioral traits in play when tailoring platform interfaces and personalized offers to ensure deeply engaged users.

Equally important is the emotional impact of wins and losses, which constitutes another key factor influencing gamblers’ decisions. A meta-analysis by (Kolomaznik et al., 2024) suggests that AI can have direct influence on users’ emotions by optimizing emotional engagement-which increases the effects of losses and wins in altering appetites for gambling. Positive emotions following a win can be reinforced by AI to encourage continued play, while negative feelings after a loss can be alleviated with the help of algorithms (Kolomaznik et al., 2024). Studies also confirm that single-framed appeals heighten users’ perceptions of gains and losses, directly influencing gambling behavior and platform retention (Kolomaznik et al., 2024). 

Collectively, the literature indicates that AI serves to amplify betting impulses (Wong et al., 2023), create personalized user experiences (Poudel et al., 2024), steer betting patterns (Chan, 2010), exacerbate problematic gambling through overlap with gaming (Gainsbury et al., 2017), and activate neural mechanisms that support rewards and dopamine responses (Zack et al., 2020). 

Taken together, the interplay of digital mechanisms and AI strategies strongly influences user behavior—shaping cognitive processes, altering emotional reactions to wins and losses, and reinforcing patterns characteristic of addiction. Building on these insights into cognitive and emotional factors, the next step is to examine how AI-driven incentives and rewards directly shape user decision-making in gambling.

### 2.3. The Influence of Technologyon User Decisions in Gambling

Although various external incentives, such as bonuses and monetary rewards, influence user behavior in gambling contexts, Homonoff (2018) argues that any incentive, regardless of its size, can significantly shape behavior, as predicted by Prospect Theory. Bonuses, as instant monetary stimuli, can increase user motivation and significantly influence immediate decisions, even when they are relatively small. Nevertheless, Schöttner (2017) shows that bonus and commission structures directly influence work behavior and motivation, concluding that bonuses generally elicit a stronger reaction than commissions. This body of research clearly demonstrates that bonus-based rewards can have a significant impact on shaping users’ long-term behavior.

Seeley et al. (2014) further support the idea that external incentives impact user behavior, demonstrating how win–loss structures influence risk perception and, in turn, user decision-making. For instance, the option to withdraw winnings early (“cash-out”) significantly affects bet size by heightening perceived control over outcomes (Seeley et al., 2014). Similarly, other studies indicate that additional features like cash-out may increase the risks users are willing to take because they offer an enhanced sense of control (Schöttner, 2017). Meanwhile, Seeley et al. (2014), drawing on game theory, show that adjustments to bonus structures lead to shifts in individual decisions, ultimately encouraging more risk-taking.

From a behavioral standpoint, decreasing the number of times a user in the betting domain bets after achieving a gain (either profit or early cash-out) could represent a risk-regulating behavior. Bounded rationality (Simon, 1955) suggests that users may want to experience the intention to stop betting once they have arrived at a satisfactory state or when emotional self-regulation systems (e.g., reducing anxiety by taking early cash-out) persuade the individual from engaging in the risky endeavor. This satisficing behavior would indicate an underlying association with previously published work surrounding goal completion, impulse control, and reward-seeking in gambling contexts (Marchica et al., 2020; M. Auer & Griffiths, 2022).

In the online wagering space, the integration of AI in targeting and personalizing incentives—such as bonuses and special offers—amplifies their effects, increasing both user participation and betting activity (Murch et al., 2024). AI-powered mechanisms not only analyze user behavior but also effectively inject and modulate certain behaviors on online gambling platforms (Murch et al., 2024).

Given the powerful influence of technology on gamblers’ behavior outlined above, regulatory frameworks have increasingly sought to address and mitigate these effects.

Building on the literature reviewed above, our theoretical contribution can be structured along three interrelated dimensions: (1) the behavioral-cognitive level, where we examine how AI-based personalization may intensify decision-making biases, such as impulsivity or perceived control; (2) the ethical dimension, where we consider the potential manipulation of user autonomy via algorithmic reinforcement strategies; and (3) the policy dimension, where we explore how these mechanisms raise concerns about transparency and accountability in digital gambling platforms.

Our theoretical framework does not advance a deterministic view of AI; it imagines personalization technologies as potential enhancers of current cognitive heuristics. The explanatory logic is based on behavioral science, not technological determinism.

### 2.4. European Regulations on AI and Online Gambling

The regulatory framework for online gambling in Europe is shaped by a comprehensive body of legislation that integrates national regulations with European directives, ensuring transparency and user protection. However, the use of artificial intelligence to contextualize user experiences and enhance retention strategies has also attracted regulatory attention. The most salient point is that the AI Act from the EU refers to gambling AI applications as “high-risk”, which subjects them to stringent transparency and algorithmic auditability requirements (Wagner et al., 2024). Simultaneously, existing European legislation establishes major prohibitions regarding the use of personal data and automated systems, including artificial intelligence (AI) in gambling. For instance, the General Data Protection Regulation (European Parliament and Council, 2016) specifies the prohibitions on automated decision-making, including profiling, when it produces legal effects or a similarly significant effect for the individual (European Parliament and Council, 2016). In addition, the Recommendation of the European Commission on online gambling facilities highlights consumer protection and responsible data use in online betting situations (European Commission, 2014). In totality, these frameworks serve to limit the use of AI in betting algorithms wherever personal data processing is concerned. The weakness in the legislation raises concerns over the protection of vulnerable users against algorithmic recommendations and their right to know how these recommendations are personalized (Brown et al., 2022).

On the national level, the regulatory approaches differ immensely. Others have interpreted the European Court of Justice’s ruling that states have the right to impose their own regulation on online gambling, so long as they comply with transparency standards and respect the principles of the European single market (Bogaert & Cuyvers, 2011). For example, in the U.K., the UK Gambling Commission proposed banning the provision of excessive algorithmic incentives to users displaying signs of problematic gambling behavior, thereby limiting the risk of addiction. Thus, while Italy and the UK have taken distinct approaches to AI integration in gambling regulations, these differences have led to conflicts between national and European legal frameworks (Laffey et al., 2016).

Furthermore, the challenge is imposed by competition between AI legislation and data protection laws. GDPR prescribes parameters of broad consent and use of data. However, there is currently no tightly knit regulatory framework clearly defining the role of AI in gambling applications. This gap is acknowledged by recent EU initiatives (e.g., the Cyber Resilience Act (Mueck et al., 2023)) that aim to bolster cybersecurity and protect consumers from technological abuses.

In conclusion, while the EU has put its laws in place to govern the functioning of AI in high-risk sectors, including gambling, legislative gaps still exist to enable algorithmic transparency, protect vulnerable users, and ensure that users have the right to know how AI-driven decisions are made and how those decisions impact them (Brown et al., 2022; Wagner et al., 2024).

## 3. Methodology

### 3.1. Statistical Models and Research Design

In this study, two datasets were used. The first one is publicly available on Kaggle and contains 50,000 records (kingabzpro, 2023). This dataset consists of bets placed on the Bustabit platform from 31 October to 10 December 2016. The other one, also found on Kaggle, consists of over 2 million recorded bets placed on Bustabit between 26 April and 30 April 2021 (Dmytro Bubela, 2023). The combined datasets allow for a comparison of user behavior across two distinct timeframes, with the 2021 data providing insight into the evolving effects of more advanced algorithmic mechanisms.

While the datasets themselves do not contain direct metadata about AI-driven interventions, our interpretation of the 2021 dataset as reflecting a more algorithmically enhanced environment is based on industry developments between 2016 and 2021. During this period, online gambling platforms, including Bustabit, expanded their use of adaptive reward systems, real-time behavioral analytics, and personalization tools that are commonly associated with AI-driven user engagement. We do not claim to observe AI mechanisms directly but rather infer their likely presence through the timing, platform evolution, and behavioral shifts observed in the data.

Because there are no internal platform logs or metadata that would offer assurance of specific algorithmic personalization events, this study is considered observational/quasi-experimental. We are relying on time comparisons (2016 and 2021) as proxies for larger changes in personalization technologies. Therefore, we regard anything we could infer about the impact of AI as indirect and exploratory.

Both datasets are drawn from Bustabit, a platform that hosts a single, continuous multiplayer gambling game classified as a game of chance. Specifically, Bustabit operates on a “crash” model in which users place a stake before a real-time multiplier begins to rise. Players must decide when to cash out, aiming to do so before the system “crashes” at a randomly determined point. The payout is proportional to the multiplier at the moment of withdrawal. This design creates a dynamic and high-variance betting environment that encourages short-term decision-making under uncertainty.

All bets analyzed in this study originate from this one probabilistic game. The dataset does not include different games or distinguish between games of skill and chance. As such, our behavioral analyses pertain solely to this uniform format.

In the absence of experimental manipulations or platform metadata that specifically linked AI use, we opted to use temporal comparisons to satisfy our comparison condition. The 2016 dataset functioned as a kind of pseudo-control condition, as it represented the time prior to when AI enhanced personalization was absent or minimally developed in gambling platforms. Conversely, the 2021 dataset reflected a later period in the development of such platforms and a wider acceptance of algorithmically driven engagement tools (e.g., real-time bonus delivery, behavioral segmentation) throughout the industry. While we cannot establish the presence of AI in particular user experiences, the year-based comparison is suitable for comparing behavior change that is consistent with more algorithmic use. 

This methodology does not allow for the direct causal inference of AI mechanisms; rather, it is a quasi-experimental view with justification rooted in the temporal separation of a baseline environment in 2016 and a temporally evolved technological community in 2021. Therefore, we should interpret all findings as exploratory, consistent with but not meant to imply in any way AI-related behavioral effects. Future work using either platform-side metadata, experimental randomization, or mixed-method triangulation would be needed to confirm any of these associations.

The analysis involved running a number of basic regression analyses using key variables detailed in Table 1. The variable “Bet” refers to the amount wagered by the user in a particular session, which we view as an indicator of the financial commitment for each round of betting. The variable “Bonus_dummy” is a binary variable that is coded as 1 if the user received a personalized bonus or promotional incentive during the session, and as 0 if they did not; this variable serves to identify when the user was experiencing specifically targeted promotional incentives. The variable “Profit” is the amount that the user earned (or lost), after accounting for their winnings and losses, from all of the bet amounts. “EarlyCashout” is also a binary variable that was created to indicate if the user cashed out of an early wager before that wagering round expired, which could indicate that the user was trying to lock in their gains or control their losses (or a combination of both). Lastly, the variable “TotalBets” represents the total number of individual bets in that session, which we interpret as an indicator of betting intensity or persistence in behavior.

They provide insight into using AI-driven mechanisms to affect betting behavior, decision-making, and user engagement. This analysis was intended to inform the subsequent section by elucidating how technology affects user decision-making through the chosen independent variables. Both OLS and panel regressions were applied to predict betting user characteristics. A comparison between the 2016 and 2021 datasets aims to reveal whether users have changed their betting habits in response to newly introduced algorithmic mechanisms on the platform. 

To gain a more comprehensive view of the factors driving betting behavior, multiple regression models were employed. The OLS regression models estimate the direct effects of bonuses, profit, and early cash-out behavior on users’ bet size and frequency. Additionally, fixed-effects panel regression was used to examine whether these effects varied between 2016 and 2021, capturing potential algorithm-induced changes in user retention and decision patterns.

Although multicollinearity diagnostics three regression models are based on considerations of relevance from theory and the data structure, the variables selected for each model were those that were interpretable in behavioral models of gambling and were accessible in the dataset. Variables, such as TotalBets, when applicable, were modeled as both predictors and outcome variables, depending on the research question for that model.

#### 3.1.1. Model 1—OLS Linear Regression

This model estimates the influence of bonuses, profit, number of bets, and early cash-out behavior on the amount wagered:

Bet = β_0_ + β_1_·Bonus_dummy + β_2_·Profit + β_3_·TotalBets + β_4_·EarlyCashout + ϵ

#### 3.1.2. Model 2—OLS Linear Regression

Here, we explore how the same set of explanatory variables influence the number of bets placed by each user:

TotalBets = β_0_ + β_1_·Bonus_dummy + β_2_·Profit + β_3_·EarlyCashout + ϵ

where

- Bet represents the amount wagered (log-transformed as log(Bet + 1));
- Bonus_dummy equals 1 if the user received a bonus, 0 otherwise;
- Profit is the net profit obtained by the user;
- TotalBets is the total number of bets placed;
- EarlyCashout equals 1 if the user performed an early cashout, 0 otherwise;
- ϵ denotes the residual error term.

#### 3.1.3. Model 3—Fixed Effects Panel Regression

To assess temporal differences in betting behavior between 2016 and 2021, we estimate a panel model with user fixed effects. The dependent variable is the bet size per session, and the model includes an indicator for year and interaction terms to capture potential shifts in user behavior attributable to changes in platform mechanisms:

Bet_it = β_0_ + β_1_·Year_it + β_2_·Bonus_dummy_it + β_3_·Profit_it + β_4_·EarlyCashout_it + u_i + ϵ_it

where

- Bet_it is the logarithm of the amount wagered by user i at time t;
- Year_it is a dummy variable (1 for 2021, with 2016 as the reference year);
- Bonus_dummy_it indicates whether the user received a bonus;
- Profit_it is the logarithm of the user’s profit;
- EarlyCashout_it indicates whether the user performed an early cashout;
- u_i represents individual fixed effects;
- ϵ_it is the idiosyncratic error term.

The employment of AI in online gambling, from a psychological perspective, can be compared with principles of operant conditioning according to (Skinner, 1938). They reinforce gambling behavior with intermittent rewards the optimal moments for intervention, as identified by the algorithm, allowing for compulsive platform use. Continual behavioral conditioning posed a significant threat to their mental well-being, a function of debt stress, anxiety, and risk-taking tendencies in the longer run (M. Griffiths, 2008; M. D. Griffiths & Auer, 2013). Such dynamics raise serious ethical concerns about the duties of platform operators and the urgent need for regulations on exploiting cognitive vulnerabilities of users.

### 3.2. Data Analysis and Assumption Testing

The data were analyzed using Stata 14.2 to produce the descriptive statistics for all study variables. The dataset contains 446,898 observations.

Table 2 shows the means, standard deviations, variances, skewness, and kurtoses for the main variables. The variable Bonus_dummy represented that a user was given some kind of a bonus and has a very low mean of 0.033, confirming that the use of this kind of incentive is rare. In contrast, EarlyCashout, which measures the tendency of users to cash out their winnings early, achieves an average of 0.516, which indicates almost an equal number of users fast cashing out and those waiting longer with their funds. User profit averages 337.57 with a very large standard deviation (5691.7), indicating an extremely high disparity in winnings. This distribution is characterized by very high skewness (101.07) and kurtosis (15,177.03), indicating a highly asymmetric outcome where a small number of users have substantially larger-than-average winnings.

Thus, given an average of 3.006 total bets per user (SD = 2.688), there are evidently considerable differences between users in this metric. The mean number of bets is 492.36, with a very high standard deviation (9493.43). The extremely high skewness (73.12) indicates that only a few users placed an exceptionally large number of bets, resulting in an uneven distribution.

To enhance the distributional characteristics of the data, natural logarithmic transformations were applied to three important variables: Profit, TotalBets, and Bet. After the transformations, these variables will be referred to as ln_Profit, ln_TotalBets, and ln_Bet (with ln_Bet equal to ln(Bet + 1) to allow for zero cases). After the transformations, the distributions of these variables were much closer to normal. It appears that the log transformations reduced the influence of outliers and stabilized the variance, making the data more amenable for linear regression.

To evaluate the seven hypotheses, we employed four complementary models in Stata 14.2. Normality checks (Shapiro–Wilk) and multicollinearity diagnostics (VIF) informed the necessary log-transformations and final specifications, and every coefficient reported in Table 3 is based on heteroscedasticity-robust standard errors.

Descriptive exploration showed that 50% of accounts placed ≤ 4 bets and 13% only one bet—evidence of a sizable recreational segment. All continuous variables with marked skewness (Bet, Profit, TotalBets) were log-transformed to stabilize variance; categorical predictors were coded 0/1. No influential outliers were detected after examining leverage and Cook’s distance.

Collectively, this analytical strategy permits (i) direct tests of H1–H6 on stake size and persistence, and (ii) an assessment of whether these relationships shifted between 2016 and 2021 (H7), thereby linking behavioral outcomes to the presumed evolution of AI-enabled platform features.

## 4. Results 

### 4.1. Effect of Early Cash-Out on Bet Size

A Welch two-sample *t*-test on the log-transformed stake per session (ln_Bet) confirmed that players who withdrew their winnings early wagered significantly more than those who waited (t = −180.06, *p* < 0.001). The early cash-out group had a mean ln_Bet of 3.15, compared to 2.34 for the late group—corresponding to a 0.81 log-unit difference. These results confirm H1 (see Table 4; summary in Table 5, row H1).

### 4.2. Determinants of Early Cash-Out

Exploratory inspection indicated that one half of all accounts placed four bets or fewer and 13% placed a single bet, underscoring a sizeable recreational cohort. A logistic-regression model predicting early cash-out included log-transformed TotalBets together with Bonus_dummy, ln_Profit, Bet, and LossStreak. Fewer total bets, receipt of a bonus, and lower profit all increased the likelihood of early withdrawal (*p* < 0.001). These patterns support H2 (bonus is associated with reduced stake sizes) and H3 (profit is linked to increased stake amounts), as summarized in Table 5 (rows H2–H3).

The logistic regression shows that users with fewer total bets are significantly more likely to cash out early (*p* < 0.001), supporting the notion that lower engagement predicts early withdrawal. Profit, bonus receipt, and recent betting losses also enter the model as significant controls (full coefficients reported in Table 4). Together, these predictors discriminate reliably between early and late cash-out decisions.

### 4.3. Predictors of Betting Persistence

A robust OLS model with ln_TotalBets as outcome (R^2^ = 0.241, *n* = 446,898) revealed that bonuses, higher profit, and early cash-out each reduced the number of consecutive bets (all β < 0, *p* < 0.001). Thus H4, H5, and H6 are all supported (Table 5, rows H4–H6). Although the three drivers share a dampening effect, their behavioral mechanisms may differ—an issue we return to in the Discussion.

### 4.4. Year-on-Year Differences in Betting Behavior (2016 vs. 2021)

A user-level fixed-effects panel model accounted for 82.5% of the variance in ln_Bet (R^2^ = 0.825). The 2021 indicator carried a negative and significant coefficient (β ≈ −0.51, *p* = 0.002), showing that average stakes per session fell relative to 2016. Interaction terms between Year 2021 and bonus, profit, or early cash-out were non-significant, indicating stable effect directions over time. Consequently, H7 is partially supported—the baseline stake changed, but the relationships in H1–H3 did not (Table 5, row H7).

In light of the fact that interaction effects are not statistically significant, we are cautious about ascribing a temporal increase to an increasing influence of the algorithm. Although the patterns are consistent with technological development more broadly, they cannot necessarily be attributed to AI-based personalization using Model 3.

## 5. Discussion

### 5.1. Early Cash-Out and Session-Level Stakes 

The *t*-test reported in Section 4 confirms that players who withdraw winnings early wager higher stakes than those who wait. The effect is sizable—an 0.81 log-unit difference in ln_Bet, equivalent to stakes 2.3 times larger in raw terms. Conceptually, “quick winners” may recalibrate their risk tolerance upward after a prompt payoff, or they may be nudged by real-time recommender systems that highlight attractive odds or bonus prompts once a cash-out is detected. Either way, AI-mediated feedback appears to extend its influence beyond the withdrawal moment and into subsequent wagering decisions.

While this study interprets larger bets following early cash-out and higher profits as indicative of gain-oriented behavior, we acknowledge that not all gambling behavior is driven by financial incentives. A substantial body of clinical and psychological literature highlights escape gambling—the use of gambling to regulate negative affect or avoid stress—as a stronger predictor of problematic gambling than the pursuit of financial gain alone. In this light, certain behavioral markers identified in our dataset (e.g., brief betting sessions, sudden withdrawal, volatility in stake sizes) may also reflect avoidance-based engagement. Although our data do not allow us to distinguish user motivations directly, it is important to consider that AI-driven personalization may amplify these patterns, especially if users engage during emotionally vulnerable periods. Future work should integrate affective or motivational data to clarify the psychological profiles that underlie the behavioral trends we observed.

### 5.2. Bonus and Profit Effects on Stake Size

Regression results (Table 5) show a symmetrical pattern: bonuses depress stake size (approximately a 49% reduction), whereas profit amplifies it. A bonus injects fresh liquidity, allowing users to experiment with lower wagers while preserving their bankroll; in contrast, profit boosts confidence and encourages the reinvestment of gains. For operators, this dichotomy implies that promotional credits temper immediate risk but may sacrifice short-term revenue per bet; for regulators, the finding highlights how different incentives steer risk-taking in opposite directions.

### 5.3. Interpreting Predictors of Betting Persistence

All three predictors—bonuses, profit, early cash-out—diminish the total number of consecutive bets. Bonuses appear to satisfy short-term engagement, reducing the need for extended play; profit activates a “quit-while-ahead” heuristic; early cash-out locks in gains and limits further exposure. Taken together, these mechanisms act as natural brakes on prolonged betting, partially counter-balancing retention features that push for longer sessions. The interplay suggests that platforms could modulate session length, deliberately or not, by calibrating bonus frequency and cash-out frictions.

### 5.4. Temporal Shifts in Betting Patterns

Between 2016 and 2021 the platform experienced a marked drop in average stake size (β ≈ −0.51 log-units). Yet the core relationships identified in H1–H3 remained stable, as Year × Predictor interactions were non-significant. This indicates that incremental improvements in AI—better timing of offers, refined risk scores—changed the baseline appetite for risk without altering the behavioral logic linking bonuses, profit, or cash-out decisions to stake size. For industry stakeholders, the result underscores how algorithmic fine-tuning can recalibrate global wagering levels while leaving relative behavioral drivers intact; for policy makers, it signals that harm-reduction thresholds may need periodic adjustment as platforms evolve.

We acknowledge the possibility that AI-driven personalization is one influencing factor for shifts in some behaviors, and that other contextual shifts may also explain these behavioral differences (for example, changes to regulations, other platforms, user demographics, and so on).

These behavioral shifts may not result solely from AI-driven personalization. Other contemporaneous factors—such as broader user adoption, increased recreational participation, or post-pandemic digital habits—may have also shaped betting outcomes. As such, we interpret the observed differences not as isolated effects of AI, but as patterns that are likely moderated by a constellation of social, cultural, and economic changes.

We interpret the shifts from 2016 to 2021 through the lens of advances in personalization technologies, but we do not claim a causal link. We examine shifts in aggregate data based on observable behavior over time, and our analysis is exploratory and hypothesis-generating, rather than confirmatory. 

Since the interaction terms between time and behavioral variables were not significant, the temporal aspect of algorithmic influence should be considered suggestive but not causal. Together, these findings underscore the need for direct evidence regarding AI personalization over time.

### 5.5. Associated Risks—AI-Induced Addictive Behavior

The results indicate that while AI can improve user experience and enhance engagement, it also poses considerable risk. Predictive systems and personalized algorithms can exacerbate players’ likelihood of developing addictive tendencies.

According to the data, AI-powered elements of gambling platforms can influence users’ behaviors when deciding to cash out or how much to bet. AI can track the exact time someone is about to log off a gambling platform and incentivize them to stay and play with personalized bonuses. This can contribute to increasing play-times and enhancing the proclivity toward disordered behaviors. The effects of reinforcement learning mechanisms may be triggered when AI identifies the most optimal times to offer a user a bonus, which heightens impulsivity and lessens the users’ conscious control of betting behaviors (Poudel et al., 2024; Wong et al., 2023). Such mechanisms can reinforce a cycle of compulsive betting, regardless of whether the user is trying to recover losses or increase winnings.

Studies have shown that the visibility of AI with better financial rewards may increase gambling propensity (Wong et al., 2023). At the same time, the role of bonuses in player behavior raises questions regarding the role of platforms offering hooking incentives. Past work suggests that AI can foster addictive behavior by continuously analyzing user data (Poudel et al., 2024). User behavior after a few losses also shows how vulnerable people can be to predictive algorithms. Players who have benefited from large winnings often seek to increase those winnings, feeding into a loop of potentially compulsive behavior. Complex neural network-based algorithms read and exploit betting patterns (Chan, 2010). Scholars note the extent of the overlap between gaming and gambling, suggesting there is also a greater likelihood of problematic behaviors when platforms offer social casino games that incorporate AI technologies (Gainsbury et al., 2017). Similarly, modern AI-driven gambling experiences intensify neurobiological processes linking uncertainty, reward, and dopamine signaling, which may in turn foster addictive behavior in vulnerable individuals (Zack et al., 2020). Given these considerations, there is a clear need for revised regulations and stricter transparency related to the use and availability of AI on platforms promoting online gambling. The highlighted themes point to a collaborative effort required from researchers, operators, and policymakers to ensure that vulnerable users are protected, while also highlighting the potential harms of AI-driven gambling.

### 5.6. Alignment with Previous Research on AI-Driven Gambling

The results of this study align with key conclusions in the specialized literature regarding the influence of artificial intelligence on user behavior in online gambling platforms. In line with (Wong et al., 2023), our analysis confirms that algorithmic mechanisms, including the transparency and visibility of AI, significantly affect users’ intent to place bets. Moreover, the observed effects on users who quickly cash out their winnings support the hypothesis that financial incentives and the perception of control, previously examined by (Seeley et al., 2014) and (Schöttner, 2017), can amplify tendencies toward more aggressive betting strategies. As it pertains to the influence of financial bonus schemes, our study indicates they can have a temporary suppressive effect on bet size consistent with a compensation effect documented in the literature (Homonoff, 2018). The bonus schemes may also, in a supporting role, help users to remain engaged with the platform by virtue of their usage (i.e., long-term involvement), which is in line with (Murch et al., 2024), who stressed how through AI algorithms, personalization and optimization of incentive engagement can prolong user involvement and total time spent on a given site. Moreover, the results related to the association between user profit and betting behavior supports existing studies, which recognize the relevance of prior outcomes, and accumulated experience, in determining future decisions (Sirola et al., 2021). The relation between increased profitability and increased confidence, and a propensity to engage in increasing betting behavior, provide additional confirmation of prior studies regarding the cognitive mechanisms associated with financial behavior decision-making (Dennis et al., 2017; Ruggeri et al., 2020). Our results on the total number of bets placed corroborate the observations of (Chan, 2010) and (Gainsbury et al., 2017) about AI algorithms’ ability to model user behavior by identifying and influencing repetitive betting patterns. The conservative behavior noted among users placing multiple bets may represent a learning process and an adaptation in risk management strategies. In the context of the “hooking” effect, our research confirms that online platform strategies significantly shape the amount of time users spend betting by offering financial incentives and managing the emotional aspects of wins and losses, consistent with the findings of (Kolomaznik et al., 2024). Consequently, AI mechanisms that optimize the emotional interaction for users can prolong their engagement and directly affect the number and frequency of bets. Finally, the comparative analysis of user behavior in 2016 and 2021 strengthens the changing role of AI in internet gambling, as reported in earlier research (M. Auer & Griffiths, 2022; Zack et al., 2020). These changes illustrate both platform- and user-level adjustments to novel algorithms, including technical adjustments and nuanced changes in decision-making, emotion, and social behaviors. Thus, our findings further validate and expand upon the existing literature while offering additional insights for researchers and policymakers involved in regulating online platforms and preventing addictive behaviors.

### 5.7. Ethical Implications of AI Personalization in Gambling

European legislation does not include specific regulation or prohibition regarding the personalization of gambling behavior through artificial intelligence in the European Commission Recommendation of 9 July 2014 on principles for the protection of consumers and players of online gambling services (European Commission, 2014), and under the General Data Protection Regulation (European Parliament and Council, 2016). Still, processing of user data to develop AI-driven personalization remains constrained by the limitations and safeguards of the data protection regime.

In 2021, the UK Gambling Commission began consulting on the regulation of excessive algorithmic incentives for users exhibiting signs of problematic gambling. At the European level, the Artificial Intelligence Act (Ernst & Young Global Ltd., 2024) classifies the use of AI in gambling as “high risk”, which will lead to more demanding regulations on transparency and the ability to audit algorithms.

Despite the existence of efforts to regulate gambling with AI, serious deficiencies remain. There are currently no clear legal protections around algorithmic transparency or a user’s “right to know” how decisions are recommended. 

Although the General Data Protection Regulation (GDPR) does not contain a dedicated article titled “right to explanation”, Article 22 has been interpreted by legal scholars as implying such a right in the context of automated decision-making. This principle is especially relevant to gambling platforms, where personalized content and betting incentives are delivered algorithmically. In such contexts, users should be able to access meaningful information about how and why algorithmic decisions are made, including what behavioral data is being used to shape their experience.

These gaps in the law protect platforms that may use algorithms to develop strategies to shape user decisions without user awareness. Perhaps the most urgent legal gap is the lack of a legal framework that protects users’ right to know as mandated in other regulated industries with respect to algorithmic explainability.

Our study’s findings show that AI actively shapes user behavior on gambling platforms, influencing bet frequency, withdrawal decisions, and the length of time spent on the platform. By personalizing financial incentives, platforms can exploit cognitive biases such as the illusion of control and the near-miss effect, promoting more intense and risk-prone betting behavior. This influence raises key ethical questions about transparency, accountability, and the practices adopted by gambling operators. For instance, customizing financial rewards and adjusting bonuses through algorithms—to keep users engaged—may heighten addictive behaviors (Poudel et al., 2024; Wong et al., 2023). Such practices raise concerns regarding the protection of vulnerable users and the fulfillment of platforms’ social responsibility.

Existing research underscores that using AI-based predictive and personalized mechanisms can magnify the risks of unsustainable gambling behaviors, especially for individuals who are psychologically or financially vulnerable (Chan, 2010; Zack et al., 2020). Consequently, platforms bear a clear ethical responsibility to leverage AI not only to optimize user engagement but also to safeguard them from developing problematic habits.

A suitable ethical structure for AI-enabled gambling is founded on three pillars. In the first pillar, platforms should employ predictive models not only to increase engagement but also to identify risky behaviors as early as possible and to protect the most vulnerable users (M. Auer & Griffiths, 2022; Poudel et al., 2024). Second, algorithms must not be designed to take advantage of players’ impulsivity or cognitive biases; in other words, the AI should not monetize moments of temporary loss of self-control (Gainsbury et al., 2017; Kolomaznik et al., 2024). Finally, transparency and explainability are a fundamental part of these practices; gamblers need to know under which circumstances an algorithm is affecting their choice, and what specific cues personalized them to receive the offers they see (Wong et al., 2023). Following these three principles, early risk detection, banning predatory design practices, and operational transparency, would lead to a more responsible and trustworthy online gambling environment, and mitigate negative outcomes that might occur from consistently capitalizing on users’ engagement with artificial intelligence.

Numerous emerging frameworks in AI governance, among them the OECD Principles on Artificial Intelligence (OECD, 2024) and the EU’s High-Level Expert Group on AI Ethics Guidelines (European Commission, 2019), emphasize best practices surrounding transparency, accountability, and human-centric design. Best practices in these frameworks must be applied to gambling platforms to mitigate the risk of technology that leverages personalization to induce further compulsive behaviors and exploit vulnerable users.

In practical implementation, platforms could enact these principles through a series of specific interventions. For instance, they could enact default betting limits for new or at-risk users to restrict excessive losses. Regular, mandated audits of algorithms should take place to ensure that personalization aspects do not exacerbate addiction or exploit vulnerabilities of users. Additionally, behavioral nudges in the form of session time alerts, trackers of spending amounts, or voluntary time-outs could be used as friction-based interventions to encourage user contemplation in decision-making. The steps mentioned would be in line with ethical AI design and would further address emerging norms associated with digital governance.

### 5.8. Practical and Research Implications

The empirical picture that emerges from hypotheses H1–H7 is deliberately ambivalent: algorithmic incentives appear capable of both restraining and amplifying gambling behavior, depending on context and timing. For platform operators, this duality is valuable because it offers a design lever. Our findings suggest that bonuses delivered at the right moment, together with low-stake prompts, can shorten sessions by reducing both average stake size and the number of consecutive bets; at the same time, early cash-out cues and profit feedback are linked to larger individual stakes, hinting at a different pathway to revenue growth. A data-driven loyalty strategy could therefore balance session length against stake size, meeting commercial targets without automatically escalating risk.

From a regulatory perspective, the year-on-year decline in average stakes illustrates how incremental algorithmic tuning can shift population-level risk profiles without altering the surface effect of familiar levers such as bonuses or profit messages. Static loss-limit thresholds risk rapid obsolescence as platforms iterate; hence, regulators will require continuous access to telemetry and algorithm-audit trails in order to recalibrate safeguards in near real-time.

The study also identifies several research opportunities. First, natural-experiment designs that exploit staggered algorithm roll-outs would identify causal effects more cleanly than the cross-sectional contrasts presented here. Second, pairing behavioral logs with psychometric or neuro-physiological data would clarify whether early cash-out and profit cues act through impulsivity, perceived control, or reinforcement scheduling. Third, comparative work across multiple platforms is essential, as Bustabit’s crash-game format may not generalize to sports-betting or casino verticals where pacing, sensory feedback, and reward contingencies differ.

Ultimately, the results suggest that platform designers and regulators should continue to consider that very small but subtle choices on the part of designers or regulators (such as manipulating the timing of a bonus, or, for instance, sending a user prompt about a new application interface) can unintentionally encourage risk-taking behavior. Furthermore, even in the absence of AI traceability, considering how users converge on paths as part of computer-mediated action can help inform a more ethical or responsible design of platforms, as well as criteria for assessing policies made based on platform dynamics.

Bonuses, often assumed to heighten risk by providing “found money,” in our data coincide with smaller stakes and fewer bets. We interpret this as a short-term compensatory response: the bonus is perceived as a partial gain, prompting a temporary reduction in risk. Yet intermittent rewards are well known to sustain engagement across sessions, so our results neither confirm nor rule out a longer-term reinforcing effect. Exploring that temporal dynamic will require longitudinal or mixed-method approaches.

Finally, any future investigation of algorithmic gambling environments should embed an ethics-by-design perspective. The very features that appear to help casual players self-limit—such as post-bonus stake reductions—can just as readily be tuned to prolong high-value play. Transparent labeling of personalized offers, opt-out mechanisms for vulnerable users, and independent audits of reinforcement algorithms represent baseline safeguards if the industry wishes to leverage AI responsibly. A collaborative agenda linking engineers, behavioral scientists, and policy makers is therefore essential to capture the upside of personalization while containing its downside risk of addiction.

### 5.9. Limitation

When interpreting the results of this study, it is important to exercise caution because of a number of methodological and contextual limitations. These may include the characteristics of the dataset, the absence of certain key variables, and other outside factors that may have impacted user behavior independently of the mechanisms under the scope of the study.

The datasets do not contain platform logs indicating whether, when, or how algorithmic personalization (e.g., bonus timing, dynamic prompts, adaptive pay-out suggestions) was delivered to individual users. As a result, we were unable to identify a specific “AI variable” to include in any regression equation, or to draw causal conclusions about the algorithmic interventions. Thus, any conclusion drawn about the influence of AI is conceptual and based on behavior tracked over time rather than observed or documented within the internal systems.

Additionally, there is no record of any log of platform-related intervention; therefore, this study should be classified as observational and quasi-experimental research, since temporal variation is used as a surrogate for broader changes in technology. The inference about artificial intelligence remains exploratory.

No demographic fields (age, gender, socio-economic status, ethnicity, gambling experience) are present in the raw data. The absence of such covariates restricts our ability to control for structural differences between cohorts and opens the possibility of unobserved heterogeneity that may confound temporal comparisons.

We were unable to distinguish whether play was driven by financial gain, emotional escape, or other motives. As prior research shows that avoidance-motivated gambling is a stronger predictor of disorder than gain-seeking, some behaviors we interpret as strategic (e.g., early cash-out) might instead reflect coping mechanisms. Future work should incorporate psychometric measures or self-report data.

The 2021 observations were collected during the COVID-19 pandemic, a period marked by social isolation, financial uncertainty, and increased digital engagement. In addition, the intervening years saw expanding legalization and normalization of online gambling, potentially broadening the user base and attracting more recreational bettors. These factors may partly explain behavioral shifts that we attribute to AI-mediated design.

Although multicollinearity diagnostics were satisfactory (mean VIF < 1.5), the behavioral interdependence among predictors (bonus, profit, total bets, early cash-out) could inflate R^2^ without clarifying causal pathways. Moreover, we did not estimate interaction terms—such as Profit × EarlyCashout or Bonus × TotalBets—to avoid overfitting in an already complex model. As a result, subtle conditional effects remain unexplored.

One limitation of this study is the absence of significant interaction effects, resulting in a weakened empirical basis for attributing changes in behavior over time to changing AI personalization. This suggests that our results should be viewed as observational and for generating hypotheses, rather than being confirmatory.

Taken together, these limitations mean that our findings should be viewed as behavioral signals consistent with—but not definitive proof of—algorithmic influence. Future work would benefit from (a) direct platform-side logs of AI interventions, (b) demographic and psychometric covariates, (c) experimental or mixed-method designs that can disentangle pandemic-era effects, and (d) interaction-aware modeling frameworks that balance explanatory power with parsimony, and (e) more granular data (e.g., platform-side A/B test logs, real-time timestamps for when bonuses were delivered, and user-level segmentation), to better isolate and confirm the behavioral impact of algorithmic interventions.

Apart from the potential for addiction, these findings may also support our broader theoretical point: that personalization at the platform level through more subtle reinforcement mechanisms might, through more subtle reinforcement mechanisms, involve a deeper engagement with cognitive heuristics like loss aversion, perceived control, or satisficing in cognitively crafting what digital addiction means. The importance of our analysis is not limited to addiction as an outcome but helps identify the ways in which technology affordances have the power to shape decision-making under uncertainty and affect user agency in a manner that is only less harmful than clinically harmful outcomes. Though perhaps less harmful than clinical harm, this type of behavioral shaping is vital to current discussions about technology and AI ethics, user autonomy, and the pressing need for proper regulatory oversight, such as considering diary methods when designing persuasive systems.

Thus, while we find features indicative of AI-enabled personalization, they remain inferential on our part and should be viewed as theoretically influenced signals indicating a relationship rather than empirically supported evidence of algorithmic causation.

## 6. Conclusions and Implications

This study demonstrates that algorithmic personalization already influences several aspects of online gambling behavior. 

Our findings identify behavioral trends that align with more algorithmic personalization than what was observed at the outset of the study. Nevertheless, without data specific to the implementation of these platforms, a conclusion about AI involvement cannot be made, and thus these findings should be interpreted accordingly.

Bonuses, profit feedback, and early cash-out options have distinct, sometimes counter-directional effects: a bonus tends to suppress immediate risk-taking, whereas profit cues and rapid withdrawal appear to encourage higher individual stakes. At population level, the average stake per session fell between 2016 and 2021 despite stable main effects for the core predictors, suggesting that incremental algorithmic tuning can recalibrate risk profiles without altering the visible levers familiar to users and regulators.

Nevertheless, it must be stated that the present study does not provide direct evidence for algorithmic interventions or platform-side modifications. The patterns observed are theorized in the context of larger technological and contextual changes that occurred between 2016 and 2021, and any mention of AI and personalization mechanisms is purely inferential.

For operators, this ambivalent pattern is commercially attractive. By calibrating the timing and magnitude of incentives, a platform can shorten sessions for some users while simultaneously raising per-stake turnover for others—thereby balancing volume, retention, and revenue. The same design flexibility, however, raises important regulatory questions. Traditional loss-limit thresholds or static bonus rules risk rapid obsolescence when reinforcement schedules can be updated in near-real time. Regulators will therefore require continuous access to telemetry streams and to auditable records of algorithm changes if they are to track and mitigate emerging harms.

The results also expose a paradox at the heart of bonus design. In our data, the moment a bonus is credited is followed by smaller stakes and fewer bets—an effect that can be interpreted as a short-term compensatory response in which a player feels partially rewarded and temporarily reduces risk. Yet intermittent rewards are well known to sustain engagement across multiple sessions, and we cannot rule out a delayed rebound once the immediate suppression dissipates. Understanding that temporal dynamic will require longitudinal or mixed-method research capable of linking event-level incentives to session-level outcomes over time.

Because the datasets lack demographic, psychological, and AI-exposure metadata, our findings remain exploratory rather than causal. The absence of statistically significant interactions between time and behavioral variables indicates that the temporal component of algorithmic influence should be regarded as suggestive, rather than a determined causal influence. The data still requires more direct evidence to substantiate causal claims about the influence of time and personalization by artificial intelligence. Nevertheless, they point to three urgent research priorities. First, staggered roll-outs of new recommender modules would create natural experiments for isolating algorithmic effects. Second, behavioral logs should be paired with psychometrics to determine whether cues operate through impulsivity, perceived control, or other motivational pathways. Third, cross-platform comparisons are necessary, as the reinforcement profile of crash games may not generalize to sports-betting or casino slots.

Finally, the ethical stakes are clear. The very features that appear to help casual users self-limit—bonus-induced stake reductions, for example—can be re-tuned to prolong high-value play. Responsible deployment therefore requires transparent labeling of personalized offers, opt-out mechanisms for vulnerable customers, and independent audits of reinforcement algorithms. Only through coordinated action among engineers, behavioral scientists, and policy makers can the benefits of AI-driven personalization be harnessed without amplifying the risks of manipulation and addiction.

In conclusion, while our research demonstrates evidence supporting the pattern of the changing effects of algorithmic systems on gambling behavior, it does not present evidence to support the direct use of AI deployment. Future studies should strive to include log-level metadata and experimental differences to disentangle the specific role of AI interventions.

## Figures and Tables

**Table 1:** Operational definitions and measurement units of studied variables.

Variable Name	Description	Unit of Measure
Id	Unique identifier for each bet	Numeric
GameID	Identifier for each gaming session	Numeric
Username	The user’s pseudonym	String
Bet	The amount wagered by the user for each session	Numeric
CashedOut	The moment when the user cashed out their winnings before a loss.	Numeric
Bonus	The amounts received by users as bonuses	Numeric
Profit	The net profit obtained by the user after each bet	Numeric
BustedAt	The moment when the gaming session ended	Numeric
PlayDate	The date and time of each bet	Datetime
EarlyCashout	Binary variable (0/1) indicating whether the user cashed out their winnings quickly (1) or delayed the withdrawal (0)	Dummy (0/1)
TotalBets	The total number of bets placed by the user on the platform	Numeric
Bonus_dummy	Binary variable (0/1) indicating whether the user received a personalized bonus or promotional incentive during the session (1 = bonus received; 0 = no bonus received)	Dummy (0/1)

**Table 2:** Descriptive statistics for key variables (2016 and 2021 combined dataset).

Variable	Mean	Std. Dev.	Variance	Skewness	Kurtosis
Bonus_dummy	0.0333	0.1795	0.0322	5.198	28.021
ln_Profit	2.136	2.3750	5.640	0.489	3.563
Profit	337.567	5691.697	3.24 × 10^7^	101.073	15,177.03
ln_TotalBets	7.206	1.666	2.776	−1.308	−1.160
TotalBets	3006.344	2688.854	7,229,935	0.486	1.708
EarlyCashout	0.515	0.4992	0.249	−0.063	1.004
ln_Bet	2.773	2.068	4.277	1.060	3.622
Bet	492.364	9493.434	9.01 × 10^7^	73.120	6744.035

**Table 3:** Regression estimates with robust diagnostics.

Hypotheses	Model	Key Diagnostics
H1	Two-sample *t*-test comparing the mean of ln_Bet between early-cash-out users (1) and late-cash-out users (0). Normality of raw Bet violated (*p* < 0.0001), hence log-transformation; post-transform Shapiro–Wilk acceptable.	*n* = 446,898; equal-variance assumption relaxed (Welch test).
H2, H3	OLS regression (Model 1) with ln_Bet as the dependent variable and Bonus_dummy and ln_Profit as focal predictors, controlling for ln_TotalBets and EarlyCashout.	VIF < 1.5 (no multicollinearity); Shapiro–Wilk on residuals significant but tolerable given large n and robust errors.
H4, H5, H6	OLS regression (Model 2) with ln_TotalBets as outcome and Bonus_dummy, ln_Profit, and EarlyCashout as predictors. Transformation reduced skewness from 73.1 to −1.3.	R^2^ = 0.241; mean VIF = 1.04.
H7	Fixed-effects panel model (Model 3), user-level, with ln_Bet as outcome, Year 2021 dummy, and all main predictors. Interaction terms Year × (Bonus, ln_Profit, EarlyCashout) were added to test moderation.	Hausman test favored fixed effects; interactions jointly non-significant (Wald, *p* > 0.10).

**Table 4:** Comparison of predictors influencing bet size and frequency (OLS and FE models).

Variabiles	Model 1	Model 2	Model 3
_cons	1.480(0.006) ***	8.022 (0.003) ***	0.832 (−0.163) ***
Bonus_dummy	−0.367 (0.007) ***	−3.582 (0.011) ***	−0.370 (0.014) ***
ln_Profit	0.787 (0.0006) ***	−0.1575294 (0.001) ***	0.771 (0.011) ***
ln_TotalBets	−0.160 (0.0007) ***		
EarlyCashout	1.517 (0.002) ***	−0.697 (0.004) ***	1.511 (0.043) ***
Year 2021			−0.508 (0.1670) **
R-squared	0.880	0.241	0.825

*** *p* < 0.01, ** *p* < 0.05. Standard errors are in parentheses. Model 1 is an OLS regression with robust standard errors. The dependent variable is the natural logarithm of the amount wagered (ln_Bet). Independent variables include a dummy indicator for bonus reception (Bonus_dummy), the log of net profit (ln_Profit), a binary variable for early cashout behavior (EarlyCashout), and the log of total bets placed (ln_TotalBets). Stata command: reg log_Bet Bonus_dummy log_Profit EarlyCashout log_TotalBets, robust. Model 2 is an OLS regression with robust standard errors. The dependent variable is the natural logarithm of the total number of bets placed (ln_TotalBets). Independent variables include Bonus_dummy, ln_Profit, and EarlyCashout. Stata command: reg log_TotalBets Bonus_dummy log_Profit EarlyCashout, robust. Model 3 is a panel data regression with fixed effects and robust standard errors. The dependent variable is ln_Bet. Independent variables include year fixed effects (i.Year), Bonus_dummy, ln_Profit, and EarlyCashout. This model controls for unobserved individual heterogeneity that is constant over time. Stata command: xtreg log_Bet i.Year Bonus_dummy log_Profit EarlyCashout, fe robust.

**Table 5:** Synopsis of hypotheses statistical tests, and empirical support.

Hypothesis	Model/Test	Predictor	Key Statistic
H1	Model 1		β = +1.517 (SE = 0.002, *p* < 0.001)
H2	Model 1	Bonus_dummy	β = −0.367 (SE = 0.007, *p* < 0.001)
H3	Model 1	ln_Profit	β = +0.787 (SE = 0.001, *p* < 0.001)
H4	Model 2	Bonus_dummy	β = −3.583 (SE = 0.011, *p* < 0.001)
H5	Model 2	ln_Profit	β = −0.158 (SE = 0.001, *p* < 0.001)
H6	Model 2	EarlyCashout	β = −0.698 (SE = 0.004, *p* < 0.001)
H7	Model 3	Year 2021 (vs. 2016)	β = −0.508 (SE = 0.167, *p* = 0.002)

## Data Availability

Publicly available datasets were analyzed in this study. These data were extracted from the following websites: https://www.kaggle.com/datasets/kingabzpro/gambling-behavior-bustabit and https://www.kaggle.com/datasets/dmytrobubela/bustabit-games (both accessed on 26 March 2025). All the information used for the analysis is available at the website mentioned above and can be accessed by creating a free account.

## References

[B1-behavsci-15-00779] Alaba-Ekpo O., Caudwell K. M., Flack M. (2024). Examining the strength of the association between problem gambling and gambling to escape. A systematic review and meta-analysis. International Journal of Mental Health and Addiction.

[B2-behavsci-15-00779] Auer M., Griffiths M. D. (2022). Predicting limit-setting behavior of gamblers using machine learning algorithms: A real-world study of Norwegian gamblers using account data. International Journal of Mental Health and Addiction.

[B3-behavsci-15-00779] Auer M. M., Griffiths M. D. (2015). The use of personalized behavioral feedback for online gamblers: An empirical study. Frontiers in Psychology.

[B4-behavsci-15-00779] Bogaert S. V. d., Cuyvers A. (2011). Money for nothing. The case law of the EU Court of Justice on the regulation of gambling. Common Market Law Review.

[B5-behavsci-15-00779] Brown R., Truby J., Ibrahim I. A. (2022). Mending lacunas in the EU’s GDPR and proposed artificial intelligence regulation. European Studies.

[B6-behavsci-15-00779] Chan V. K. Y. (2010). Using neural networks to model the behavior and decisions of gamblers, in particular, cyber-gamblers. Journal of Gambling Studies.

[B7-behavsci-15-00779] Chen C., Zhang K. Z. K., Gong X., Lee M. (2019). Dual mechanisms of reinforcement reward and habit in driving smartphone addiction: The role of smartphone features. Internet Research.

[B8-behavsci-15-00779] Clark L., Zack M. (2023). Engineered highs: Reward variability and frequency as potential prerequisites of behavioural addiction. Addictive Behaviors.

[B9-behavsci-15-00779] Dennis C. B., Davis T. D., Chang J., McAllister C. (2017). Psychological vulnerability and gambling in later life. Journal of Gerontological Social Work.

[B10-behavsci-15-00779] Dmytro Bubela (2023). Bustabit games.

[B11-behavsci-15-00779] Ernst, Young Global Ltd. (2024). The EU AI act: New rules for trustworthy AI in Europe.

[B12-behavsci-15-00779] European Commission (2014). Commission recommendation of 9 July 2014 on principles for the protection of consumers and players of online gambling services and for the prevention of minors from gambling online (2014/478/EU).

[B13-behavsci-15-00779] European Commission (2019). Ethics guidelines for trustworthy AI. High-level expert group on artificial intelligence.

[B14-behavsci-15-00779] European Parliament and Council (2016). Regulation (EU) 2016/679 of the European parliament and of the council of 27 April 2016 on the protection of natural persons with regard to the processing of personal data and on the free movement of such data, and repealing directive 95/46/EC (general data protection regulation).

[B15-behavsci-15-00779] Flack M., Morris M. (2015). Problem gambling: One for the money…?. Journal of Gambling Studies.

[B16-behavsci-15-00779] Gainsbury S. M., King D. L., Russell A. M. T., Delfabbro P., Hing N. (2017). Virtual addictions: An examination of problematic social casino game use among at-risk gamblers. Addictive Behaviors.

[B17-behavsci-15-00779] Griffiths M., Viren M. (2008). Problem gambling and European lotteries. Gaming in the new market environment.

[B18-behavsci-15-00779] Griffiths M. D., Auer M. (2013). The irrelevancy of game-type in the acquisition, development, and maintenance of problem gambling. Frontiers in Psychology.

[B19-behavsci-15-00779] Homonoff T. A. (2018). Can small incentives have large effects? The impact of taxes versus bonuses on disposable bag use. American Economic Journal: Economic Policy.

[B20-behavsci-15-00779] Iordache D.-M., Mihai F., Aleca O. (2024). Two-decade bibliometric analysis of collaborative trends in gambling studies: A structured literature review and analysis of gambling research (2004–2023). The International Conference on Economics and Social Sciences.

[B21-behavsci-15-00779] Ivanov K. (2021). Betting and lottery business–policies, taxes and benefits. University Economic Bulletin.

[B22-behavsci-15-00779] Junaedi J. (2024). England is the Largest Center for Online Gambling Activity in the World, Versus Indonesia is Exposed to Online Gambling Emergency Stage Five. International Journal of Law, Crime and Justice.

[B23-behavsci-15-00779] kingabzpro (2023). Gambling behavior bustabit.

[B24-behavsci-15-00779] Kolomaznik M., Petrik V., Slama M., Jurik V. (2024). The role of socio-emotional attributes in enhancing human-AI collaboration. Frontiers in Psychology.

[B25-behavsci-15-00779] Laffey D., Della Sala V., Laffey K. (2016). Patriot games: The regulation of online gambling in the European Union. Journal of European Public Policy.

[B26-behavsci-15-00779] Lee H.-P., Chae P. K., Lee H.-S., Kim Y.-K. (2007). The five-factor gambling motivation model. Psychiatry Research.

[B27-behavsci-15-00779] Marchica L. A., Keough M. T., Montreuil T. C., Derevensky J. L. (2020). Emotion regulation interacts with gambling motives to predict problem gambling among emerging adults. Addictive Behaviors.

[B28-behavsci-15-00779] Mattson R. E., MacKillop J., Castelda B. A., Anderson E. J., Donovick P. J. (2008). The factor structure of gambling-related cognitions in an undergraduate university sample. Journal of Psychopathology and Behavioral Assessment.

[B29-behavsci-15-00779] Mestre-Bach G., Steward T., Granero R., Fernández-Aranda F., Talón-Navarro M. T., Cuquerella À., Baño M., Moragas L., del Pino-Gutiérrez A., Aymamí N., Gómez-Peña M., Mallorquí-Bagué N., Vintró-Alcaraz C., Magaña P., Menchón J. M., Jiménez-Murcia S. (2018). Gambling and impulsivity traits: A recipe for criminal behavior?. Frontiers in Psychiatry.

[B30-behavsci-15-00779] Mueck M. D., On A. E. B., Du Boispean S. (2023). Upcoming European regulations on artificial intelligence and cybersecurity. IEEE Communications Magazine.

[B32-behavsci-15-00779] Murch W. S., Kairouz S., Dauphinais S., Picard E., Costes J.-M., French M. (2023). Using machine learning to retrospectively predict self-reported gambling problems in Quebec. Addiction.

[B31-behavsci-15-00779] Murch W. S., Kairouz S., French M. (2024). Establishing the temporal stability of machine learning models that detect online gambling-related harms. Computers in Human Behavior Reports.

[B33-behavsci-15-00779] OECD (2024). Recommendation of the council on artificial intelligence.

[B34-behavsci-15-00779] Parveen N., Ahsen S., Hassaan H. M., Mirza M., Iqbal S. (2024). The psychological and behavioral mechanisms of online gambling game addiction: A comparative study of cognitive biases, reward systems, and intervention strategies. Journal of Policy Research.

[B35-behavsci-15-00779] Polyzoidis P. (2019). “Young slaves in the land of gambling”: The state as a helpless viewer of the struggle of the youth with gambling. American International Journal of Social Science.

[B36-behavsci-15-00779] Poudel S., Choudhari J., Panta N., Kumar H., Leszkowitz D., Ahmed S. S. (2024). Revolutionizing addiction medicine: The role of artificial intelligence. European Psychiatry.

[B37-behavsci-15-00779] Redish A. D. (2004). Addiction as a computational process gone awry. Science.

[B38-behavsci-15-00779] Ruggeri K., Alí S., Berge M. L., Bertoldo G., Bjørndal L. D., Cortijos-Bernabeu A., Davison C., Demić E., Esteban-Serna C., Friedemann M., Gibson S. P., Jarke H., Karakasheva R., Khorrami P. R., Kveder J., Andersen T. L., Lofthus I. S., McGill L., Nieto A. E., Folke T. (2020). Replicating patterns of prospect theory for decision under risk. Nature Human Behaviour.

[B39-behavsci-15-00779] Schöttner A. (2017). Optimal sales force compensation in dynamic settings: Commissions vs. bonuses. Management Science.

[B40-behavsci-15-00779] Seeley C. J., Cashaback J. G. A., Smith C. T., Beninger R. J. (2014). Altering the shape of punishment distributions affects decision making in a modified iowa gambling task. Journal of Behavioral Decision Making.

[B41-behavsci-15-00779] Simon H. A. (1955). A behavioral model of rational choice. The Quarterly Journal of Economics.

[B42-behavsci-15-00779] Sirola A., Kaakinen M., Savolainen I., Paek H.-J., Zych I., Oksanen A. (2021). Online identities and social influence in social media gambling exposure: A four-country study on young people. Telematics and Informatics.

[B43-behavsci-15-00779] Skinner B. F. (1938). The behavior of organisms an experimental.

[B44-behavsci-15-00779] van Holst R. J., Chase H. W., Clark L. (2014). Striatal connectivity changes following gambling wins and near-misses: Associations with gambling severity. NeuroImage: Clinical.

[B45-behavsci-15-00779] Wagner M., Borg M., Runeson P. (2024). Navigating the upcoming European Union AI act. IEEE Software.

[B46-behavsci-15-00779] Whiteford S., Hoon A. E., James R., Tunney R., Dymond S. (2022). Quantile regression analysis of in-play betting in a large online gambling dataset. Computers in Human Behavior Reports.

[B47-behavsci-15-00779] Wong I. A., Chau K. F., Chan H. U. (2023). An empirical study on customers’ gambling intention in AI-supported casinos. Journal of Hospitality and Tourism Technology.

[B48-behavsci-15-00779] Zack M., St. George R., Clark L. (2020). Dopaminergic signaling of uncertainty and the aetiology of gambling addiction. Progress in Neuro-Psychopharmacology and Biological Psychiatry.

